# Effect of Ocular Movements during Eye Movement Desensitization and Reprocessing (EMDR) Therapy: A Near-Infrared Spectroscopy Study

**DOI:** 10.1371/journal.pone.0164379

**Published:** 2016-10-26

**Authors:** Daniele Rimini, Filippo Molinari, William Liboni, Marina Balbo, Roberta Darò, Erika Viotti, Isabel Fernandez

**Affiliations:** 1 Biolab, Department of Electronics and Telecommunication, Politecnico di Torino, Torino, Italy; 2 “Un Passo Insieme” ONLUS Foundation, Valdellatorre, Torino, Italy; 3 EMDR Italy Association, Bovisio Masciago (MB), Italy; Tokai University, JAPAN

## Abstract

**Introduction:**

Eye Movement Desensitization and Reprocessing (EMDR) is a psychotherapeutic treatment resolving emotional distress caused by traumatic events. With EMDR, information processing is facilitated by eye movements (EM) during the recall of a traumatic memory (RECALL). The aim of this study is to investigate the effects of ocular movements of EMDR on the hemodynamics of the prefrontal cortex (PFC).

**Material and Methods:**

Two groups were recruited: a trial group (wEM) received a complete EMDR treatment, whereas a control group (woEM) received a therapy without EM. PFC hemodynamics was monitored by near-infrared spectroscopy during RECALL and during focusing on the worst image of the trauma (pre-RECALL). The parameters of oxy- (oxy-Hb), and deoxy-hemoglobin (deoxy-Hb) were acquired and analyzed in time domain, by calculating the slope within pre-RECALL and RECALL periods, and in the frequency domain, by calculating the mean power of oxy-Hb and deoxy-Hb in the very-low frequency (VLF, 20–40 mHz) and low frequency (LF, 40–140 mHz) bandwidths. We compared pre-RECALL with RECALL periods within subjects, and pre-RECALL and RECALL parameters of wEM with the corresponding of woEM.

**Results:**

An effect of group on mean slope of oxy-Hb and deoxy-Hb in pre-RECALL and oxy-Hb in RECALL periods was observed. wEM showed a lower percentage of positive angular coefficients during pre-RECALL with respect to RECALL, on the opposite of woEM. In the frequency domain, wEM had significant difference in oxy-Hb and deoxy-Hb LF of left hemisphere, whereas woEM showed no difference.

**Discussion and Conclusion:**

We observed the effect of EM on PFC oxygenation during EMDR, since wEM subjects showed a mean increase of oxy-Hb during RECALL and a decrease during pre-RECALL, as opposed to woEM. Frequency analysis evidenced a reduction of activity of sympathetic nervous system in wEM group during pre-RECALL. Our outcomes revealed a different hemodynamics induced by eye movements in wEM with respect to woEM group.

## Introduction

Eye Movement Desensitization and Reprocessing (EMDR) is a psychotherapeutic treatment approach that facilitates resolution of emotional distress and symptoms due to traumatic and disturbing life experiences. It adopts a standardized protocol and it is recommended in the management of stress syndromes, such as post-traumatic stress disorders (PTSD)[1][2]. EMDR is based on the adaptive information processing: some events occurring in childhood, young-adult age, and during the entire lifespan, can cause a strong emotional impact, blocking the ability of the central nervous system of adequately processing information. Furthermore, traumatic events are dysfunctionally stored in memory, containing related emotions, perceptions, cognitions, as well as the disturbing physical sensations. Since the event-related information is not properly managed, subject can perceive discomfort, potentially leading to the onset of illnesses. The objective of EMDR therapy is the recovery of the natural information streaming, in order to reach an adaptive resolution [3]. The natural mechanism of cerebral information processing is resumed by the use of alternate and rhythmic stimulation of cerebral hemispheres, obtained by means of eye movements (EM), interleaved by the recalling of the memory itself and its cognitive, behavioral and somatic elements (RECALL)[4].

To objectively explore the physiological effects of EMDR, functional imaging techniques, such as nuclear imaging [5], electroencephalography (EEG)[6], and functional Magnetic Resonance Imaging [7], have been exploited. Modifications in the patterns of cerebral activity have been evidenced, particularly in those brain regions involved in stress management: the so-called hypothalamus-pituitary-adrenal (HPA) axis (an articulated response network to a stressful condition which involves deep brain regions, such as hypothalamus and amygdala) and cortical areas, such as the prefrontal cortex (PFC) [8]. Nevertheless, the bias of the aforementioned effects remains unresolved, since all subjects were submitted to the therapy, generally in pre- and post-treatment conditions.

A way to overcome this bias would be to put in evidence the effect of ocular movements characterizing the EMDR protocol, by comparing a testing group performing the treatment with respect to a control group without eye movements. Some specific studies aimed at exploring the difference between the use of EMDR with and without eye movements. Engelhard *et al*. showed that eye movement causes a decrease in arousal, flexibility in attention and memory processing, as well as an improved semantic recalling [9]. Lee and Cuijpers performed a meta-analysis of 15 clinical trials comparing EMDR with Vs. without eye movement and pointed out the beneficial effects of eye movement [10]. The limitations of these studies regarded the use of self-reported measures as indicators of performance and the absence of a functional comparison of therapy with and without eye movement.

As EMDR is a dynamic protocol in which the patient is asked to switch from eye movements to giving feedback after the set of eye movements, it is necessary to assess adequately the cerebral implication of the EMDR protocol by means of high temporal resolution neuroimaging techniques. The Near-Infrared Spectroscopy (NIRS) is a non-invasive functional technique that monitors cerebral metabolism and hemodynamics by measuring relative concentrations of oxy-hemoglobin (oxy-Hb) and deoxy-hemoglobin (deoxy-Hb) in the brain. It is based on an infrared light intensity absorption measurement that reflects the different oxygenation condition of the underlying tissue [11]. NIRS is currently used to investigate cognitive processes [12], to differentiate pathological conditions [13], and to localize cerebral lesions [14]. The main advantage of NIRS is that it monitors effectively PFC microcirculation, allowing the inspection of autoregulation and neuronal activity in a non-invasive and real-time way. NIRS was recently used to document a reduction in the PFC oxygenation during the RECALL period [15].

The aim of this study is to examine the effect of eye movement in PFC activity during an EMDR session, in the attempt of filling gaps of previous studies, focusing only on the local concentration and temporal modification of oxy-hemoglobin, without further exploring the complex mechanism of cerebral autoregulation induced by a response to EMDR stimulation. To achieve our goal, two patient groups underwent an EMDR session: the first group received complete EMDR therapy with EM, whereas the second (control) group received the same EMDR Protocol without the EM.

## Materials and Methods

We acquired the NIRS signals during the EMDR treatment of two groups. The first group performed the complete EMDR protocol (i.e. with EM) and will be indicated as wEM. The second control group performed the EMDR protocol without eye movements and will be named as woEM.

### Participants and inclusion criteria

Twenty-one patients were consecutively recruited among the ones treated at “Un passo insieme” that accepted to participate to the study. Subjects were randomly divided in two groups: one group (wEM), constituted by 11 subjects (mean age 33.3 ± 6.34 2 males, 9 females), and a second one (woEM) constituted by 10 subjects (mean age 31.8 ± 5.60 5 males, 5 females). All subjects were preliminary assessed with a battery of self-administered psychological tests: an Impact of Event Score (IES) [16], a Symptom Checklist-90 (SCL-90) [17], and a Test Evaluation Checklist (TEC). Afterwards, a major trauma for each subject was chosen. Two levels of severity were defined: trauma of extreme stress (T), referring to all the events which may threaten life of the subject, such as natural disaster, or car accident, and trauma (t) referring to relational trauma, such as family conflicts, separations, betrayals, bullying. The allocation of each subject to either “T” or the “t” group was based on clinical history.

All patients were informed about the aim of the study and were aware that their therapy session would have been recorded and all of them signed an informed consent. The study received the approval by the Ethical Committee of the EMDR Italy Association.

### EMDR Protocol

The EMDR protocol is an integrative eight-phase approach having patients recall distressing images while receiving, bilateral sensory input, including side to side eye movements. The procedure was carried out by a trained psychotherapist and the 8 phases are the following:

1. HISTORY TAKING:
  This first phase is meant to obtain background information and identify suitability for EMDR treatment. Therapist identifies processing targets from events in patient’s life according to the standardized protocol. Procedures include standard history taking questionnaires and diagnostic psychometrics.
2. PREPARATION:
  Therapist helps patient create a safe place and prepare patient for EMDR processing of targets. This involves education regarding the symptom picture, as well as explain metaphors and techniques that foster stabilization and a sense of personal control.
3. ASSESSMENT:
  Access the target for EMDR processing by stimulating primary aspects of the memory. This involves eliciting the image, negative belief currently held, desired positive belief, current emotion, and physical sensation and baseline measures.
4. DESENSITIZATION:
  Therapist helps client processes experiences toward adaptive resolution (no distress). This incorporates eye movements that allow the spontaneous emergence of insights, physical sensations and other memories.
5. INSTALLATION:
  Help increase connections of positive cognitive networks. This can be done by enhancing the validity of the desired positive belief and fully integrate within the memory network.
6. BODY SCAN:
  Complete processing of any residual disturbance. Help patient concentrate on and process any residual physical sensations.
7. CLOSURE:
  Ensure patient’s stability at the completion of an EMDR therapy session and between sessions. Provide patient with self-control techniques if needed and brief patient regarding expectations and behavioral reports between sessions.
8. REASSESSMENT:
  Ensure maintenance of therapeutic outcomes and stability of patient. Therapist evaluates effects and integration within the larger social system.

All the subjects received the entire EMDR protocol. Within phase 4 (DESENSITIZATION), we compared between the wEM and woEM groups the RECALL phases in which the subjects were recalling the event *per se* as well as the phase in which they were asked to focus on the worst image on the event itself (pre-RECALL): along with eye movements in wEM groups and without eye movements in the woEM group.

### NIRS data recording and processing

The NIRS is based on specific characteristics of the human tissues and of the hemoglobin. The near-infrared (NIR) light (wavelength range approximately 650—950nm) is poorly absorbed by human tissues, and this allows to easily penetrate through skin. The NIR is not blocked by the skull and thus it is used to monitor brain functions [18]. The NIR light interacts with biological tissues, as it is absorbed by pigmented compounds, called chromophores, or scattered in the tissues. The principal source of attenuation is given by hemoglobin whose absorption spectrum depends on its oxidized/reduced state. Furthermore, greatest NIR light absorption is due to hemoglobin circulating in small vessels (< 1 mm of diameter) of microcirculation, such as capillaries, arteriole, and venules, whereas it is scarcely sensitive to big vessels [19]. Due to concentration of oxy-Hb and deoxy-Hb are sensitive indicators of changes in cerebral blood flow (CBF)[20], and since CBF is an index of neuronal activity and neurovascular coupling [21], NIRS is a useful tool for neuroimaging of human brain.

In a NIRS recording system, an infrared light beam is injected into the skull by a photo—emitter placed on the scalp; a photo—detector, placed few centimeters far from the emitter, acquires the photons emerging from the skull. By means of the Beer—Lambert law, attenuation changes at a given wavelength are measured, and relative changes in concentration of oxy-Hb and deoxy-Hb are quantified [22]. Furthermore, because of the scattering, the light no longer travels as a straight line, hence the light pathlength becomes longer than the physical transmission length. As a consequence, even assuming no light absorption, only some of the incident light can reach the opposite side and be detected as the transmitted light [23].

In our study, we used a commercially available NIRS device (NIRO-200NX, Hamamatsu Photonics K. K., Japan) equipped by two probes. Each probe consisted of a photo—emitter and three infrared LED photo—detectors (peak wavelength nominally equal to 735, 810, and 850 nm). If the physical distance (d) is longer than approximately 3 cm, the light pathlength (L) is proportional to d and the constant of proportion is called the Differential Pathlength Factor, or DPF (L = DPF∙d). The DPF for the adult skull has the typical value of 6.26 [24]. Before probes application, subject's frontal skin was carefully cleaned, in order to remove the thin lipid film, which may reflect back part of the NIR energy. Probes were positioned bilaterally on the subject's forehead 2 cm away from midline and 1 cm above the supraorbital ridge, with detector placed medially with respect to the emitter. Relative concentration of oxy-Hb and deoxy-Hb were continuously measured. In addition, with the adopted NIRS device, Total Oxygenation Index (TOI), and Tissue Hemoglobin Index (THI) were acquired: they provide, respectively, the percentage ratio of oxygenated to total hemoglobin, and a measure of relative value of total hemoglobin. TOI provides information about mixed arterial—venous saturation, whereas THI is representative of cerebral vasomotor reactivity. NIRS data were recorded at a sampling rate of 2 Hz, and then digitally transmitted to a PC laptop at the rate of 192 kB/s.

In order to correctly separate the pre-RECALL from RECALL events in the NIRS acquisitions, each EMDR session was recorded by a digital video camera (DCS-900, *D-LINK Corporation*, *Taiwan*) placed behind the therapist and aligned to the patient’s head, so that the eye movements were properly recorded while patient was tracking therapist’s hand. To recognize them, we synchronized the NIRS signal to Video recordings and the events were pointed out by therapist’s indications.

NIRS data were preprocessed with a low-pass filtering with a 16^th^ order Chebyshev filter with a cut-off frequency of 250 mHz, followed by a fifth-order high-pass Chebyshev filter at 25 mHz. Eventually, computed signals were analyzed in time and frequency domains by custom developed routines running in Matlab (TheMathworks Inc., Natick, MA, USA).

### Time domain

Initially, for each subject, we identified the beginning of pre-RECALL and RECALL events from the recorded video and utilized them to separate the NIRS signals in the corresponding phases. Within each identified period, the curve slopes were calculated: a positive angular coefficient represented an increase of concentration of the corresponding parameter, whereas a negative coefficient meant a decrease. When all slopes were computed, the mean percentage of positive coefficients was determined for each subject.

### Frequency domain

NIRS data were analyzed in frequency domain as well, since spontaneous oscillations overlapping the cerebral hemodynamic signal can be revealed and effectively characterized by NIRS, and a functional stimulus causes a modulation in the amplitude of LF and VLF oscillations [25]. Spectral components can be mainly divided into two band classes: the Very Low Frequency (VLF), also known as B-waves, whose spectrum goes from 20 to 40 mHz, and the Low Frequency (LF), called M-waves, ranging from 40 to 140 mHz. On the one hand, VLF is thought to be generated by brainstem nuclei modulating small vessels lumen, whereas LF reflects systemic oscillations of arterials pressure and they are modulated by sympathetic nervous system [26]. Since frequency components of NIRS signal vary in time, they cannot be considered as stationary: for this reason, they were analyzed by a time-frequency distribution, rather than by a spectral analysis based on Fourier transform. NIRS data were processed with a time-frequency distribution *D*_*xx*_(*t*, *f*) belonging to Cohen's class, and the Choi-Williams (CW) distribution was used as kernel with selectivity set to 0.5 to preserve a suitable compromise between lower attenuation of interference terms, and cleaner representation. A complete description of the custom developed time-frequency toolbox and of the processing technique is available [27]. Briefly, this toolbox firstly computes the instantaneous autocorrelation function of a time series *x*[*n*], then obtains the corresponding ambiguity function by an inverse Fourier transform, applies the CW kernel, and finally obtains the *D*_*xx*_(*t*, *f*) by a double Fourier transform from the lags to the time and frequency variables. Once the time-frequency representations of the signals are obtained, the percentage distribution of power in VLF and LF bands were calculated for oxy-Hb and deoxy-Hb in right and left hemispheres within each pre-RECALL and RECALL period.

### Statistical analysis

Overall, the correlation between trauma severity levels and previous EMDR experiences with NIRS parameters were evaluated by performing a Pearson’s Chi-Square test and computing the correlation ratio (η). Then, time parameters were analyzed to understand the main effects of eye movement on brain oxygenation. Firstly, we compared mean slope changes of oxy-Hb, deoxy-Hb, TOI, and THI during pre-RECALL/RECALL periods for wEM and woEM groups: for each parameter, a 2-way analysis of variance (ANOVA) was carried out, considering group (wEM, woEM) and hemisphere (right, left) as factors, and number of pre-RECALL/RECALL periods, trauma severity (t or T), and previous EMDR experiences (present or absent) as covariates. Further analysis on the percentage of positive coefficients was carried out only for those variables that showed a statistically significant difference in previous ANOVA. Subsequently, a two samples t-test was made to compare the difference of percentage of positive angular coefficients between wEM and woEM groups, which may be interpreted as a difference within the EMDR sessions’ trends. Finally, we performed a paired t-test to compare the different percentage of positive slopes of pre-RECALL/RECALL periods within the two groups. Moreover, since multiple parameters were acquired with the same device, it was plausible to consider them correlated within each subject: consequently, to take into account intercorrelations and to provide an overall synthesis of hidden details in multidimensional dataset, a multivariate analysis of variance (MANOVA) of time parameters was accomplished. Mean Angular coefficients for both right and left sides for oxy-Hb, deoxy-Hb, TOI and THI of pre-RECALL and RECALL periods were included in the analysis.

As for angular coefficients, frequency components were compared at a subject as well as at a group level. The former was achieved by a paired t-test to compare spectral power of VLF and LF of pre-RECALL and RECALL; the latter by a two-sample t-tests of VLF and LF in pre-RECALL and RECALL between wEM and woEM groups.

For all tests, significance level was set at 0.05, and, since the two groups had different numerosity, distinct variance was assumed.

## Results

Two subjects (1 belonging to the wEM group and 1 woEM) were excluded from the analysis due to bad quality of NIRS registrations.

Table 1 summarizes the demographics of the remaining 10 wEM and 9 woEM subjects. No significant Pearson’s Chi-Square coefficients (p > 0.1) and correlation ratio (η < 0.32) were found.

**Table 1:** Summary demographic data. Trauma severity has been categorized as low (t) or severe (T).

Case	Group	Sex	Age	Previous EMDR treatment	Trauma severity	Number of pre-RECALL—RECALL periods
1	wEM	F	40	No	t	20
2	wEM	F	25	Yes	T	25
3	wEM	F	34	No	T	8
4	wEM	F	41	No	T	6
5	wEM	F	24	Yes	T	13
6	wEM	F	37	No	T	9
7	wEM	F	31	Yes	t	17
8	wEM	F	41	No	T	22
9	wEM	F	29	Yes	t	12
10	wEM	M	31	Yes	t	16
11	woEM	F	29	Yes	T	8
12	woEM	F	25	Yes	t	5
13	woEM	M	34	Yes	T	27
14	woEM	M	42	Yes	t	13
15	woEM	F	31	No	T	12
16	woEM	M	35	Yes	t	21
17	woEM	F	38	No	T	16
18	woEM	M	28	No	T	8
19	woEM	M	25	Yes	t	11

### wEM—woEM group comparison

**Fig 1:**
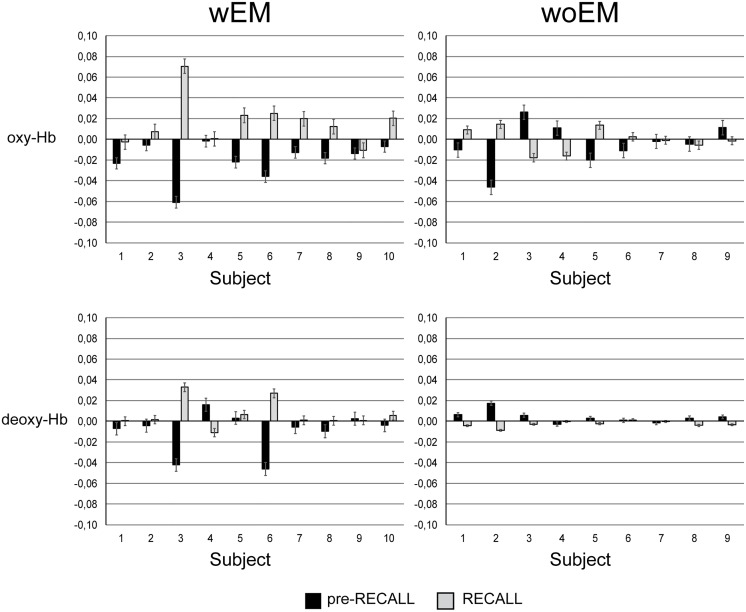
Mean slope of oxy-Hb and deoxy-Hb concentration in right hemisphere of every subject of wEM and woEM groups.

Fig 1 shows the mean values of slope of oxy-Hb and deoxy-Hb of all subjects ‘right hemisphere. It can be observed that in nearly all wEM subjects oxy-Hb slightly decreased during pre-RECALL, whereas it increased in RECALL events. Particularly, in 7 subjects on 10, oxy-Hb diminished during pre-RECALL and it increased during RECALL events. By contrast, in woEM a weak increase of about 0.4% of deoxy-Hb was observed during pre-RECALL, subsequently a decrease of about 0.27% in subsequent RECALL. Visual inspection of mean slopes was confirmed by 2-way ANOVA results (Table 2): it showed a significant main effect of group on the mean slope of oxy-Hb and deoxy-Hb in pre-RECALL periods, respectively F (1,37) = 7.600, P = 0.010, and F = 6.195, P = 0.018. As for the RECALL events, ANOVA revealed a significant effect of group on the angular coefficient of oxy-Hb (F = 7.274, P = 0.011), and deoxy-Hb (F = 7.763, P < 0.01). There was no further significant effect for hemisphere, or interaction between group and hemisphere.

**Table 2:** 2-way ANOVA of mean values of angular coefficients. Group (wEM, woEM) and hemisphere (right, left) were chosen as factors. Number of pre-RECALL/RECALL periods, trauma severity, and previous EMDR sessions were set as covariates.

Parameter	Event	*Group effect*		*Hemisphere effect*		*Interaction*	
		F	P	F	P	F	P
oxy-Hb	pre-RECALL	7.600	0.010*	0.762	0.389	0.009	0.926
	RECALL	7.274	0.011*	0.577	0.453	0.045	0.833
deoxy-Hb	pre-RECALL	6.195	0.018*	0.024	0.879	0.206	0.653
	RECALL	7.763	0.009*	0.004	0.950	0.032	0.858
TOI	pre-RECALL	0.010	0.920	2.249	0.144	0.011	0.916
	RECALL	0.012	0.913	3.904	0.057	0.007	0.933
THI	pre-RECALL	0.284	0.598	0.869	0.358	0.887	0.354
	RECALL	0.001	0.976	0.529	0.473	0.367	0.549

* P < 0.05

Since only oxy-Hb and deoxy-Hb showed significant differences in slope between wEM and woEM groups, we explored if they reported a repeatable trend within pre-RECALL and RECALL periods. Then, we computed the percentage of positive angular coefficients for these two signals. Since no significant effect of hemisphere was founded, further univariate statistical analyses were performed on the averaged values of left and right hemispheres. We checked if within wEM group the concentration of oxy-Hb or deoxy-Hb increased more frequently than woEM for both the pre-RECALL and RECALL periods. Table 3 summarizes the results. Two major observations can be done: 1) woEM subjects have a significant higher percentage of deoxy-Hb positive angular coefficients during pre-RECALL than wEM subjects (Student’s t-test, t = -2.413, P = 0.027); 2) on the opposite of RECALL, where woEM subjects have a significant lower percentage of positive angular coefficients with respect to wEM group (t = 2.654, P = 0.018). In woEM patients, the deoxy-Hb increased during the simulated pre-RECALL more frequently than wEM subjects did, whereas during RECALL the mean concentration of deoxy-Hb slightly went down.

**Table 3:** Two-sample t-test of mean percentage of positive slopes of wEM and woEM groups.

	Event	Stat t	Df	Mean	Std. Error	95% Confidence interval		Sig.
						Lower	Upper	
oxy-Hb	pre-RECALL	-1.03	9.35	-10.01	9.71	-31.84	11.82	0.33
	RECALL	1.32	16.54	9.86	7.48	-5.96	25.68	0.21
deoxy-Hb	pre-RECALL	-2.413	17.923	-21.48	8.90	-40.19	-2.77	0.027*
	RECALL	2.654	14.461	19.28	7.27	3.74	34.81	0.018*

* P < 0.05

### pre-RECALL -RECALL periods comparison

**Fig 2:**
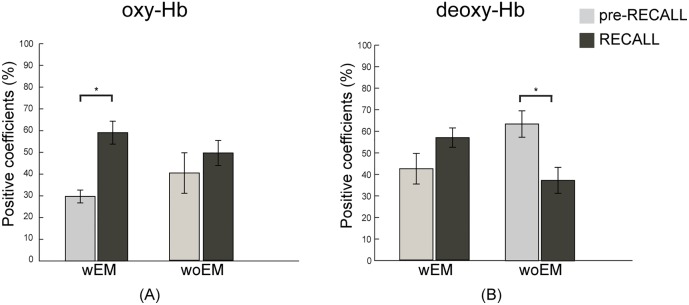
Mean percentage of positive angular coefficients of oxy-Hb (A) and deoxy-Hb (B) in pre-RECALL and RECALL periods for wEM and woEM groups. * P < 0.05.

Results of the paired t-test of mean percentage positive angular coefficients are showed in Table 4. wEM subjects showed a significant percentage of positive coefficient of oxy-Hb in pre-RECALL inferior with respect to RECALL (27.26% versus 57.16%, P < 0.002), at the contrary of woEM group (34.24% versus 46.76%, P > 0.4). By contrast, woEM subjects have a percentage of positive coefficient of deoxy-Hb in pre-RECALL significatively higher with respect to RECALL (68.02% versus 33.96%, P < 0.03), differently from wEM group (43.79% versus 55.32%, P = 0.39). Mean percentage of positive coefficients for each group and condition are showed in Fig 2.

**Table 4:** Paired t-test of mean percentage of positive coefficients in pre-RECALL and RECALL periods for wEM and woEM groups.

	wEM				woEM			
	oxy-Hb		deoxy-Hb		oxy-Hb		deoxy-Hb	
	*pre-RECALL*	*RECALL*	*pre-RECALL*	*RECALL*	*pre-RECALL*	*RECALL*	*pre-RECALL*	*RECALL*
Mean	27.260	57.160	43.790	55.320	34.244	46.767	68.022	33.967
Stat t	-4.235		-0.896		-0.824		2.628	
P	0.002*		0.394		0.434		0.030*	

* P < 0.05

### Multivariate Analysis

**Fig 3:**
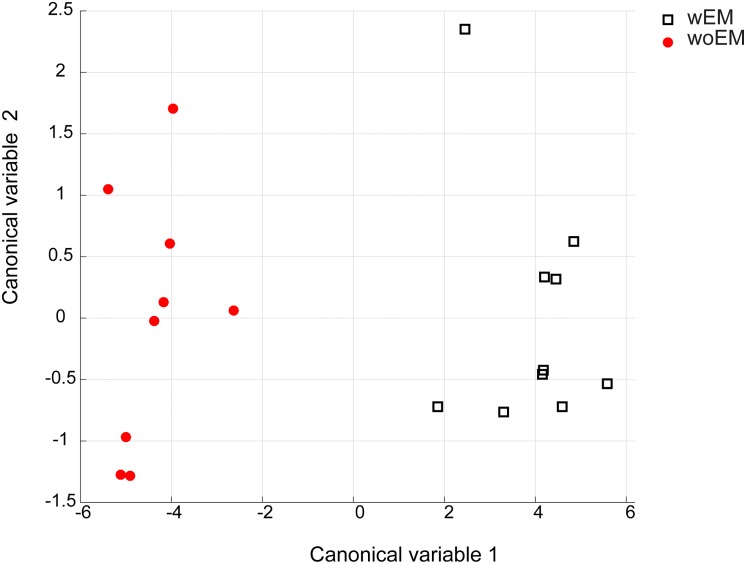
Representation of the subjects in the space of the first two canonical variables obtained by MANOVA for the wEM (square) and woEM group (circles). The first canonical variable can separate the two groups.

MANOVA allowed properly separating the two multivariate dataset which lie in a space of dimension 1. Representation of subjects as a function of the first canonical variable is reported in Fig 3, where a clear separation between wEM—woEM groups can be observed.

### Frequency analysis

pre-RECALL/RECALL paired t-tests results are reported in Tables 5 and 6 for wEM and woEM groups respectively. Overall, we observed in both wEM and woEM that VLF average power was higher during pre-RECALL than RECALL, and, consequently, LF moved to the opposite direction. Particularly, wEM subjects revealed a strong difference on mean VLF for both oxy-Hb and deoxy-Hb. However, left LF contribution of woEM showed no significant difference between pre-RECALL and RECALL periods. Summarizing, VLF was significantly different for both hemispheres in wEM and woEM groups. On the contrary, LF were significantly different for wEM, right and left hemispheres, whereas in woEM group no significant difference in left hemisphere was achieved. As for the comparison of frequency components of wEM with woEM, no significant differences were found for VLF and LF of oxy-Hb and deoxy-Hb.

**Table 5:** Paired t-test results of percentage of spectral power in VLF and LF bandwidths in pre-RECALL and RECALL periods for wEM group (mean ± standard deviation).

wEM						
			*pre-RECALL*	*RECALL*	*T Stat*	*P value*
**Right**	*VLF*	oxy-Hb	38.2(±0.3)	35.6(±0.3)	6.0908	0.0002*
		deoxy-Hb	36.7(±0.3)	34.2(±0.3)	5.5141	0.0004*
	*LF*	oxy-Hb	49.1(±0.2)	50.3(±0.2)	-3.173	0.01*
		deoxy-Hb	51.5(±0.4)	52.4(±0.4)	-2.793	0.021*
**Left**	*VLF*	oxy-Hb	38.8(±0.5)	35.8(±0.5)	7.861	<0.000*
		deoxy-Hb	39.9(±0.5)	36.8(±0.5)	6.670	<0.000*
	*LF*	oxy-Hb	48.0(±0.0)	49.5(±0.2)	-4.710	0.001*
		deoxy-Hb	48.0(±0.3)	49.6(±0.2)	-4.152	0.002*

**Table 6:** Paired t-test results of percentage of spectral power in VLF and LF bandwidths in pre-RECALL and RECALL periods for woEM group (mean ± standard deviation).

woEM						
			*pre-RECALL*	*RECALL*	*T Stat*	*P value*
**Right**	*VLF*	oxy-Hb	35.9(±1.6)	33.3(±1.4)	4.314	0.003*
		deoxy-Hb	36.0(±0.4)	33.9(±0.3)	2.469	0.04*
	*LF*	oxy-Hb	51.4(±1.3)	52.7(±1.0)	-2.745	0.025*
		deoxy-Hb	50.4(±0.3)	51.3(±0.2)	-1.343	0.216
**Left**	*VLF*	oxy-Hb	36.9(±1.1)	34.7(±1.1)	3.274	0.011*
		deoxy-Hb	39.0(±0.5)	37.5(±0.6)	1.890	0.095
	*LF*	oxy-Hb	51.2(±1.2)	52.0(±1.2)	1.860	0.182
		deoxy-Hb	48.5(±0.3)	48.7(±0.3)	1.860	0.848

* P < 0.05

## Discussion

Our results showed a different oxygenation of PFC area in the case of EMDR therapy with ocular movements with respect to a control group without pre-RECALL. On the one hand, in woEM was observed a mean increase in deoxy-Hb during pre-RECALL, compensated by a decrease during RECALL. By contrast, wEM subjects showed an increase of oxy-Hb during RECALL, whereas pre-RECALL was characterized by a decrease of concentration of oxy-Hb. This different behavior is confirmed by the fact that, by comparing the two groups, mean risen of deoxy-Hb in pre-RECALL is markedly more evident than wEM. Mainly, by considering mean variations of all acquired NIRS parameters, the two groups clearly showed different underlying patterns.

Several functional studies have been performed in order to study effects of EMDR on brain cerebral activation. Pagani et al. demonstrated a pattern of resynchronization of cerebral EEG waves, and an increase of interhemispheric connectivity during EMDR sessions [6]. Nuclear medicine was adopted to look into the CBF by comparing cerebral activation during exposure to the traumatic memory before and after therapy. It was showed a significant modification of neuropsychological condition and CBF determined by EMDR therapy in several areas involved in stress management belonging to HPA axis or the neurobiology of stress diseases such as PTSD [28]. However, to the best of our knowledge, EMDR was investigated only in one study with NIRS: in Othani et al., differently from the present work, multiple EMDR sessions were performed to look over the effect of therapy over time, whereas our work was limited to a single session. However, it was observed improvement in brain perfusion for both patients with and without eye movement, so no clear association between EMDR and lateral PFC over-activity over the time was explicated. For against, our protocol provided for the recruitment of a sham group to whom therapy were submitted without eye movements, whereas in the previous study all the subjects were submitted to complete EMDR therapy. Moreover, the study protocol we adopted, allowed us to understand the effect of eye movements in subjects with traumatic memories compared to a control group, as well as understand their different physiological underlying mechanisms.

A first result of interest of the present study was the observation of a slight decrease of PFC activity in the control group during the phase without EM, brought out by the variation in deoxy-Hb concentration. This phenomenon has been yet reported in literature and it is generally defined as negative BOLD response (NBR)[29]. Several studies observed this behavior of cerebrovascular coupling in both fMRI and NIRS studies, and, since it is a not fully understood phenomenon, hypothesis about underlying physiological mechanisms are still debated [30]. One of the most reliable is that some brain regions require a raising in oxygen consumption due to the increase of their activity, then, to satisfy its supply, it is taken from contiguous areas, where NBR is observed. In our recorded cases, slight but significant NBR in PFC has been recognized, maybe due to the raising of activity in closer brain areas which probably required a supplement consumption of oxygen. It is worth to observe that during the phase without EM, woEM subjects were not focusing on a particular activity, so the resting state was theoretically the only activated network. Nevertheless, subjects could feel in a stressful condition: while recalling the event, a traumatic memory emerged without resolution, and this could lead to the alteration of the network at rest. By contrast, wEM subjects showed an opposite behavior: focusing the attention on a secondary activity with EM, allowed to diminish patient’s anxiety. The reduction in hardship was expressed in a differential modification of oxy-Hb in PFC, since it increased significantly during RECALL, then decreased to lower values during pre-RECALL: therefore, by focusing on the particular activity of eye movement, activity shifts toward the areas highlighted by previous functional studies.

The frequency domain analysis revealed that wEM subjects showed a lower LF spectral power during the pre-RECALL phase than RECALL, whereas no difference between phases was registered in woEM sample. Since LF may be linked to variability in sympathetic nervous system, eye movements effect in reducing its activity. These outcomes are consistent with those obtained by previous studies, where a reduction in sympathetic activity [31] and mostly an increase in parasympathetic [32] during pre-RECALL were reported, reinforcing the hypothesis that eye movements are dearousing.

Eventually, by adopting NIRS imaging technique to assess modification in PFC oxygenation during submission of EMDR, brain activity was non-invasively examined. Furthermore, methods we adopted to summarize data, allowed to differentiate the physiological behavior between the two groups. On the one hand, considering signals’ slope within a selected period rather than focusing our analysis only on relative mean concentrations, explained minimal modifications over time, such as those regarding the deoxy-Hb, generally difficult to characterize into a clear pattern. On the other hand, in spite of some parameters considered on their own did not significantly differ between groups, overall measurements of brain activity, including those related to mixed arterial—venous saturation and cerebral vasomotricity, represented respectively by TOI and THI, allowed a clear discrimination between the two groups. Trauma severity and previous experience of some patients with EMDR protocol may be a limitation to the study. However, the samples were designed homogeneous, and no significant correlation with measured data was observed. Altogether, NIRS provided several reliable quantitative measures of brain activity during EMDR, allowing understanding the key effects of eye movements in the therapy.

## Conclusion

We submitted two groups of subjects to EMDR therapy with and without eye movement respectively in order to observe the effects of cerebral hemodynamics, by assessing PFC with NIRS. Our outcomes revealed a different oxygenation pattern between pre-RECALL and RECALL periods in wEM group which is not observable in woEM, revealing a different hemodynamics induced by eye movements and helpful in resolving the stressful condition of trauma recalling. Our results confirmed the importance of eye movements in the framework of EMDR treatment. Eye movements were correlated with a reduced oxy-Hb concentration, which may be linked to a reduced working activity of PFC.

## Supporting Information

S1 File Raw data tables. The following raw data are provided: 1) **Demographic data**. The following information is provided for each subject: group, sex, age, previous experience with EMDR treatment (Yes or No), trauma severity and number of pre-RECALL/RECALL periods. Trauma severity was set to low (t) or high (T) levels. 2) **Angular coefficients**. Mean values of angular coefficients within pre-RECALL and RECALL periods for oxy-Hb, deoxy-Hb, TOI, and THI parameters. 3) **Positive coefficients**. Mean percentage of positive angular coefficients within pre-RECALL and RECALL periods for oxy-Hb and deoxy-Hb parameters. 4) **Frequency domain**. Mean power at the VLF (20–40 mHz) and LF (40–100 mHz) within pre-RECALL and RECALL intervals for oxy-Hb and deoxy-Hb parameters.
(XLSX)
